# Understanding the regulatory mechanisms of milk production using integrative transcriptomic and proteomic analyses: improving inefficient utilization of crop by-products as forage in dairy industry

**DOI:** 10.1186/s12864-018-4808-5

**Published:** 2018-05-29

**Authors:** Wenting Dai, Quanjuan Wang, Fengqi Zhao, Jianxin Liu, Hongyun Liu

**Affiliations:** 1 Institute of Dairy Science, College of Animal Sciences, Zhejiang University, Hang Zhou, 310058 People’s Republic of China; 2 Laboratory of Lactation and Metabolic Physiology, Department of Animal and Veterinary Sciences, University of Vermont, Burlington, VT 05405 USA

**Keywords:** Dairy cow, Forage source, Mammary gland, Milk protein production, Proteomics, Transcriptomics

## Abstract

**Background:**

Bovine milk is an important nutrient source for humans. Forage plays a vital role in dairy husbandry via affecting milk quality and quantity. However, the differences in mammary metabolism of dairy cows fed different forages remain elucidated. In this study, we utilized transcriptomic RNA-seq and iTRAQ proteomic techniques to investigate and integrate the differences of molecular pathways and biological processes in the mammary tissues collected from 12 lactating cows fed corn stover (CS, low-quality, *n* = 6) and alfalfa hay (AH, high-quality, *n* = 6).

**Results:**

A total of 1631 differentially expressed genes (DEGs; 1046 up-regulated and 585 down-regulated) and 346 differentially expressed proteins (DEPs; 138 increased and 208 decreased) were detected in the mammary glands between the CS- and AH-fed animals. Expression patterns of 33 DEPs (18 increased and 15 decreased) were consistent with the expression of their mRNAs. Compared with the mammary gland of AH-fed cows, the marked expression changes found in the mammary gland of CS group were for genes involved in reduced mammary growth/development (COL4A2, MAPK3, IKBKB, LGALS3), less oxidative phosphorylation (ATPsynGL, ATP6VOA1, ATP5H, ATP6VOD1, NDUFC1), enhanced lipid uptake/metabolism (SLC27A6, FABP4, SOD2, ACADM, ACAT1, IDH1, SCP2, ECHDC1), more active fatty acid beta-oxidation (HMGCS1), less amino acid/protein transport (SLC38A2, SLC7A8, RAB5a, VPS18), reduced protein translation (RPS6, RPS12, RPS16, RPS19, RPS20, RPS27), more proteasome- (PSMC2, PSMC6, PSMD14, PSMA2, PSMA3) and ubiquitin-mediated protein degradation (UBE2B, UBE2H, KLHL9, HSPH1, DNAJA1 and CACYBP), and more protein disassembly-related enzymes (SEC63, DNAJC3, DNAJB1, DNAJB11 and DNAJC12).

**Conclusion:**

Our results indicate that the lower milk production in the CS-fed dairy cows compared with the AH-fed cows was associated with a network of mammary gene expression changes, importantly, the prime factors include decreased energy metabolism, attenuated protein synthesis, enhanced protein degradation, and the lower mammary cell growth. The present study provides insights into the effects of the varying quality of forages on mammary metabolisms, which can help the improvement of strategies in feeding dairy cows with CS-based diet.

**Electronic supplementary material:**

The online version of this article (10.1186/s12864-018-4808-5) contains supplementary material, which is available to authorized users.

## Background

Forage accounts for 50% or more of the diet of dairy cows [1]. Therefore, the quality of forage has a large effect on bovine milk production in the dairy industry. In China, alfalfa hay (AH) is widely used as a high-quality forage, but approximately 230 million kilogram of AH has to be imported from other countries annually because of a shortage [2], substantially increasing milk production cost. Furthermore, the gap between supply and demand for high-quality forage is increasing by approximately 10% annually [3]. However, China produces approximately 100 million tons of corn stover (CS; a main crop byproduct produced in North China) each year, and most of this crop byproduct is disposed or burned [4]. The use of CS in dairy feed has been limited due to its low nutritional value. From an environmental prospective and to reduce the dependence on imported AH, it is strategically important for China to explore how to efficiently utilize these low quality forages in dairy production. Therefore, it is our goal to investigate the regulatory mechanism through which various forages exert their effects on dairy production, which will help us to develop new methods to improve the efficiency of utilizing low-quality forages (such as CS) in dairy feeding.

Compared with AH, CS has lower protein content (crude protein, rumen degradable protein, and rumen ungradable protein) and non-fiber carbohydrates [5]. In recent studies from our group, cows fed AH-based diets had better production performance in milk yield, milk protein content, and milk efficiency (milk yield/dry matter intake) compared to cows fed CS-based diets [6]. To explore the mechanisms underlying the low production performance of cows fed a CS diet, we performed the following studies. First, we found that the dairy cows fed an AH-based diet had enhanced metabolisms of several amino acids, including phenylalanine, serine, threonine, tyrosine, arginine and proline, in the mammary gland compared to cows consuming CS-based diets [7]. Second, we demonstrated that low feed and nitrogen efficiency may play a vital role in contributing to low milk protein production in the mammary glands of cows consuming low-quality forage of CS and rice straw [8]. However, the regulatory mechanisms by which the different-quality forages affect bovine mammary metabolisms and the subsequent milk production remained largely unknown.

Sustained developments in the nucleotide sequencing technology, especially RNA-sequencing, has resulted in an explosive growth in the number and quality of transcriptome sequenced for various tissues in both prokaryotes and eukaryotes [9, 10] and their potential changes under different conditions. Also, a certain of dynamicity by post-transcriptional and translational regulations of gene expression can be estimated by measuring its proteome (expressed protein set from genome) [9]. To date, iTRAQ-based quantitative proteomics has extensively improved protein identification coverage, thus providing more comprehensive linking of proteins to their metabolic functions. Recently, integrating transcriptomic and quantitative proteomic analyses have been widely used to promote a better understanding of the molecular mechanisms driving biological process in cells and tissues [11–15]. Here our objective was to elucidate a more complete understanding of molecular mechanisms underlying mammary gland adaptation to the alternative nutrient supplies provided by two rations with different forage sources (AH and CS). We performed transcriptomic and proteomic analyses of the mammary gland tissue samples collected from 12 lactating cows fed either AH) or CS).

## Methods

### Experiment design and sample collection

All animal care and procedures in this study were approved by the Animal Care Committee, Zhejiang University (Hangzhou, P. R. China) and were in accordance with the Zhejiang University’s guidelines for animal research. Twelve multiparous Chinese Holstein dairy cows were randomly assigned into two blocks, among which six cows with the milk yield of 30.3 ± 5.1 kg/d (mean ± SD) and the body weight of 604 ± 37.4 kg were fed CS as forage, and the other six cows with the milk yield of 29.2 ± 4.4 kg/d and the body weight of 608 ± 41.4 kg were fed AH as forage. The two blocks of cows were fed 55% concentrate (dry matter, DM), 15% corn silage, and either 30% CS (CS group; *n* = 6) or 23% AH and 7% Chinese wild rye hay (AH group; *n* = 6). The dietary compositions and nutritional values of both diets were reported previously [6]. The experiment was conducted over a 14-wk period, with the first 2 wk. for dietary adaptation, in Hangjiang Dairy Farm (Hangzhou, China). The cows were slaughtered at the end of experiment, and mammary tissue samples were collected from each cow, placed in sterile tubes, immediately snap-frozen in liquid N_2_, and subsequently stored at − 80 °Cfor extraction of RNA and protein.

### RNA preparation, cDNA synthesis and RNA sequencing

**Fig. 1:**
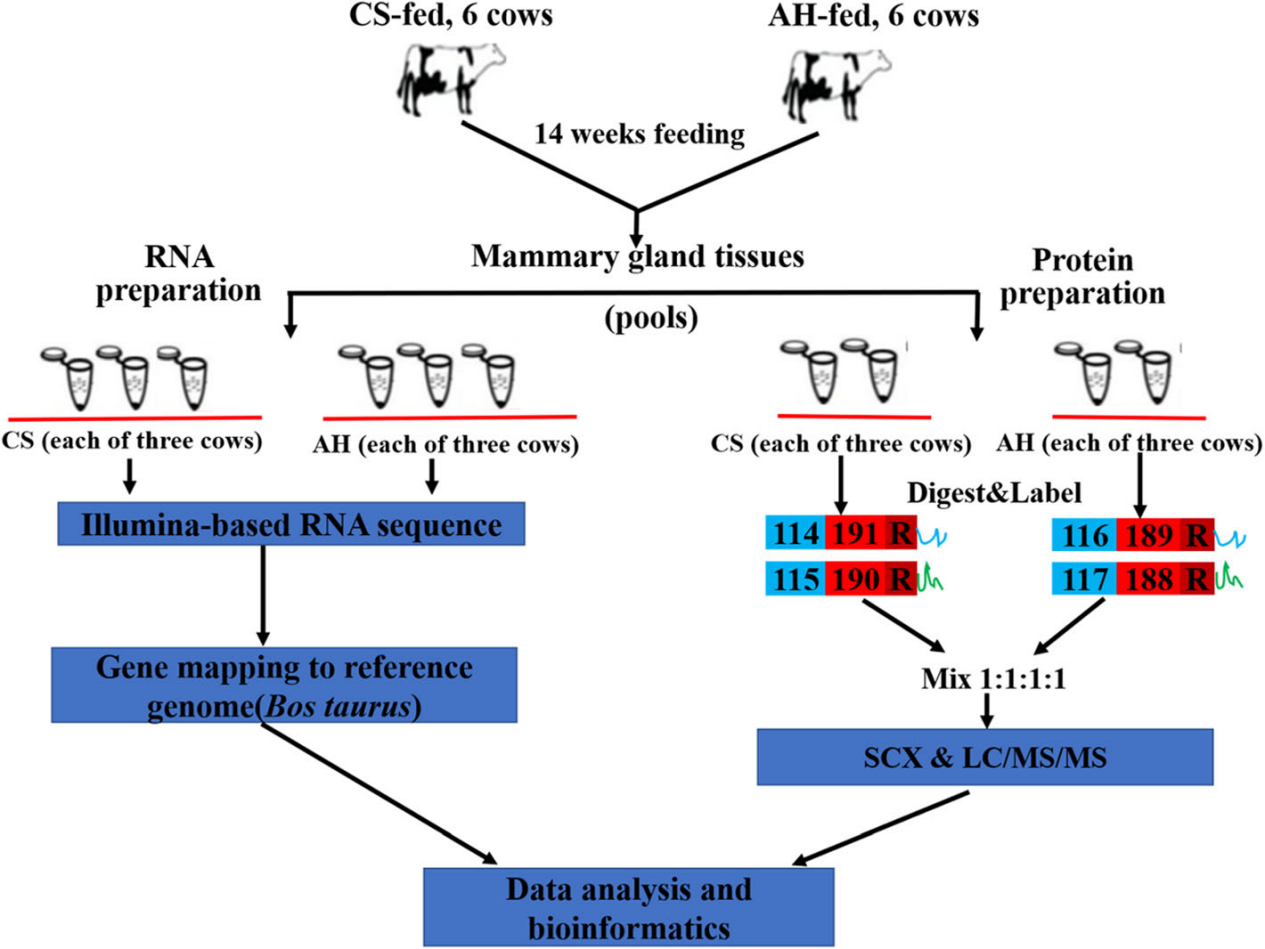
An overview of the transcriptomic and proteomic experiment. Schematic diagram of workflow of the RNA-seq transcriptomic and iTRAQ-based proteomic experiments. Six cows were fed either corn stover (CS) or alfalfa hay (AH) as forage for 14 weeks. RNA from two cows and protein samples from three cows within each group were pooled for transcriptomic (3 replicates/group) and proteomic (2 replicates/group) analyses. For transcriptomic assay, the pooled RNA was sequenced on the Illumina platform, and subsequently the reads were aligned and mapped to the *Bos taurus* genome. For the proteomic assay, the extracted proteins were digested with trypsin, and the peptides are labeled with different iTRAQ reagents, which contain reporter groups of different masses (114, 115, 116, 117), balance groups of different masses (191, 190, 189, 188), and a reactive group (R). The labeled peptides are then mixed equivalently and fractionated by strong cation exchange (SCX) chromatography. Fractions were separated by liquid chromatography (LC) and analyzed by two-step mass spectrometry (MS)

RNA was extracted from mammary tissue with Trizol reagent (Invitrogen, Carlsbad, CA, USA) and purified with Qiagen RNeasy kit (Qiagen, Valencia, CA, USA). RNA quality was examined with the Agilent 2100 Bioanalyzer (Agilent Technologies, Palo Alto, CA), and RNA quantity was measured with a NanoDrop (NanoDrop Technologies, Inc. Wilmington, DE, USA). The RNA integrity number (RIN) [16] was more than 7.0. Approximately 5 μg of high quality RNA was used for cDNA library construction according to the Illumina RNA ligation-based method [17]. 2.5 μg RNA from the mammary gland of two cows in each group were combined (three biological replicates per group; Fig. 1), and RNA sequencing was performed using a HISeq 2000 sequencing system (Illumina, San Diego, CA, USA) from LC Sciences (Houston, TX, USA). Reads were processed with Bowtie version 0.12.7 (http://bowtie-bio.sourceforge.net), aligned in Tophat version 1.3.2 (http://ccb.jhu.edu/software/tophat/index.shtml), and then mapped to bovine reference genome UMD3.1 (ftp://ftp.ensembl.org/pub/release-79/fasta/bos_taurus/dna/) [18]. Gene abundance was estimated as reads per kilobase of exon model per million mapped reads (RPKM) with Cufflinks version 1.2.1 as previously described [17, 18]. Genes with a cut-off of 1.5-fold expression changes and *p*-value less than 0.05 between CS and AH groups were defined as differentially expressed.

### The qRT-PCR analysis

Total RNA extracted from the mammary gland was reverse transcribed for cDNA synthesis using a PrimeScript^RT^ Reagent Kit with gDNA Eraser (Takara, Tokyo, Ostu, Japan) following the manufacturer’s instructions. The qRT-PCR was performed in triplicate using the Applied Biosystems 7500 real-time PCR system (Applied Biosystems, Foster City, CA, USA). Each 20 μL reaction included 50 ng of reverse transcription product, 40 nM of each forward and reverse primer [Additional file 1: Table S1, designed by Primer 5 software (Premier Biosoft International, Palo Alto, CA, USA)], and SYBR Premix Taq (Takara). The PCR program was one cycle of 95 °C for 30 s plus 40 cycles of amplification at 95 °C for 5 s and 58 °C for 30 s, followed by an additional 15 s at 95 °C, 1 min at 60 °C, and 15 s at 95 °C to generate melt curves. The relative gene expression values were calculated by the 2^−ΔΔCt^ method [19]. The gene expression levels were normalized against the internal control genes β-actin and GAPDH.

### Protein preparation and digestion

Protein preparation and digestion were performed as in the previous studies [20, 21]. Briefly, 500 mg mammary tissue was ground to a fine powder in liquid N_2_, lysed with the lysis buffer A (7 M Urea, 2 M Thiourea, 4% CHAPS (3-[(3-Cholamidopropy) dimethylammonio] propane-sulfonate), 40 mM Tris-HCl, pH 8.5), and reduced with 10 mM DTT at 56 °C for 1 h, followed by alkylation with 55 mM IAM (Iodoacetamide) in a darkroom for 1 h. The reduced and alkylated protein mixtures were precipitated by adding 4 × volume of chilled acetone and incubating at − 20 °C overnight. After centrifugation at 4 °C and 30,000×g, the pellet was dissolved in 0.5 M TEAB (Triethylamine borane; Applied Biosystems, Milan, Italy) and sonicated on ice. After centrifugation at 30,000×g at 4 °C again, an aliquot of the supernatant was assigned for determination of protein concentration by the Bradford method [22]. The proteins in the supernatant were kept at − 80 °C for further analysis.

### The iTRAQ labeling and strong cationic exchange (SCX) fractionation

Total protein (150 μg obtained by mixing 50 μg protein from the mammary glands of three cows in each group, two biological replicates per group; Fig. 1) was digested with Trypsin Gold (Promega, Madison, WI, USA) at 37 °C for 16 h with the ratio of protein: trypsin = 30: 1. After digestion, peptides were dried by vacuum centrifugation. Peptides were then reconstituted in 0.5 M TEAB and processed following the manufacturer’s protocol for 4-plex iTRAQ reagent (Applied Biosystems) [21]. The protein samples from the CS and AH groups were labeled with iTRAQ reagents 114, 115, 116 and 117. Strong cationic exchange chromatography was performed with an LC-20AB HPLC pump system (Shimadzu, Kyoto, Japan). The procedures for SCX fractionation including the elution were essentially the same as in the study of Meng et al. [20]. Finally, the eluted peptides were pooled into 20 fractions, desalted with a Strata X C18 column (Phenomenex, Torrance, CA, USA), and vacuum-dried.

### Liquid chromatography–tandem mass spectrometry (LC/MS) analysis

The sample fractions described above were further separated and identified on an LC-20 AD nano-HPLC system (Shimadzu) loaded with Q-Extractive mass spectrometer (Thermo Fisher Scientific, San Jose, CA, USA). Buffer C consisted of 2% acetonitrile (ACN) and 0.1% formic acid (FA) in Milli-Q water; buffer D consisted of 98% ACN and 0.1% FA. After resuspension with buffer C, 10 μL sample supernatant was loaded by the auto-sampler onto a C18 trap column (2 cm × 100 μm, 5 μm) and then separated on the reverse-phase analytical C18 column (100 mm × 75 μm, 3 μm). The samples were loaded at 8 μL/min for 4 min, then a 44 min gradient was run at 300 nL/min starting from 2 to 35% buffer D, followed by 2 min linear gradient to 80% buffer D, and then maintenance at 80% buffer D for 4 min, and finally return to 5% buffer D in 1 min.

Peptide analysis was performed with a Q-Exactive mass spectrometer in a positive ion mode with a selected mass range of 350–2000 mass/charge (m/z). The electrospray voltage applied was 1.6 kV. For MS scans, the m/z scan range was 350 to 2000 Da. For MS/MS scans, the m/z scan range was 100–1800. MS/MS data was acquired using the top 15 most abundant precursor ions with the ion count more than 20,000 in the MS scan. These were selected with an isolation window of 2 m/z and were fragmented via high energy collisional dissociation under normalized collision energies of 30 eV. For the MS scan, the resolving power was set to 70,000 at m/z 200, the maximal ion injection time was 10 ms and, dynamic exclusion of the selected precursor ions was 15 s. Automatic gain control (AGC) was used to optimize the spectra generated from the orbitrap, and the AGC target value was 3,000,000 for full MS and 100,000 for MS_2_, respectively. For the MS/MS scans, the resolving power was set to 17,500 at m/z 200; maximum ion injection times for MS/MS scans were at 60 ms; and the underfill ratio was defined as 0.1%.

### Protein identification and quantification

The raw files were first merged and transformed to an MGF file with Proteome Discoverer ver. 1.2 (Thermo Fisher Scientific, San Jose, CA, USA) and were then probed on the Mascot search engine (ver. 2.3.02; Matrix Science, London, UK) of the Uniprot database of bovine (*Bos tauru*) with 31,661 entries. For protein identification, the parameters were set the same as in the study by Yang et al. [23] with some minor changes: carbamidomethyl (C), iTRAQ 4-plex (N-term), and iTRAQ 4-plex (K) were defined as fixed modifications; Gln→pyro-Glu (N-term Q), oxidation (M), deamidated (NQ) were the possible variable modifications. The decoy database pattern was considered as the reverse of the target database. The method of peptide identification by false discovery rate (FDR) was performed as Sheng et al. described [24].

Relative quantification of the identified proteins was performed with the Proteome Discoverer software described above and calculated by the weighted and normalized ratios of uniquely identified peptides that belong to the specific individual protein. The integration window tolerance of the peak was set to 20 ppm. Statistics analysis was conducted using Fisher’s test. Proteins with a cutoff of 1.2-fold change between CS and AH samples and *p* < 0.05 were determined as significantly differentially expressed proteins.

### Bioinformatics analysis

Functional annotations were performed using Blast2GO program against the non-redundant protein database (NR; NCBI). Metabolic pathway analysis was conducted using R software (R version 3.2.3) according to the Kyoto Encyclopedia of Genes and Genomes (KEGG) pathway database (http://www.genome.jp/kegg/). The pathway enrichment statistics were performed by Fisher’s exact test with a *p-*value ≤ 0.05 considered as significant.

### Western blot analysis

Approximately 40 μg protein per sample was separated on 12% SDS (sodium dodecyl sulfate) polyacrylamide gels. Proteins were transferred onto 0.45 μm PVDF membranes (IPVH00010; Millipore, Boston, Massachusetts, USA) and blocked with blocking buffer (Beyotime, Jiangsu, China). The membranes were incubated with primary antibodies to SCP2 (non-specific lipid-transfer protein 2, ab140126; Abcam, Cambridge, MA, USA), IDH2 (isocitrate dehydrogenase 2; ab131263, Abcam), SLC7A8 [(solute carrier family 7 (amino acid transporter, L system), member 8, ab75610, Abcam)], COL4A2 (collagen, type IV, alpha 2, sc-70,243, Santa Cruz biotechnology; Cambridge, MA, USA), and β-actin (Beyotime). After washing with TBST [tris-buffered saline containing 0.02% (*v*/v) Tween-20] three times, the membranes were incubated with goat anti-rabbit IgG or goat anti-mouse IgG secondary antibodies conjugated with horseradish peroxidase (Beyotime), incubated with ECL (electrochemiluminescence) Western Blotting Substrate Kits (Beyotime), and finally visualized with a Kodak Image Station 2000MM (Kodak Molecular Imaging Systems, New Haven, USA). The relative intensities of bands were calculated with ImagePro Plus 6.0 software (Media Cybernetics, Washington, MD, USA) using β-actin as the reference protein.

### Statistical analysis

The data on relative mRNA expression by qRT-PCR and protein expression by Western blot were analyzed through one-way ANOVA in SAS (SAS 9.0). The statistical significance was declared at *p* ≤ 0.05.

## Results

### Overview of transcriptomic and quantitative proteomic analyses

**Fig. 2:**
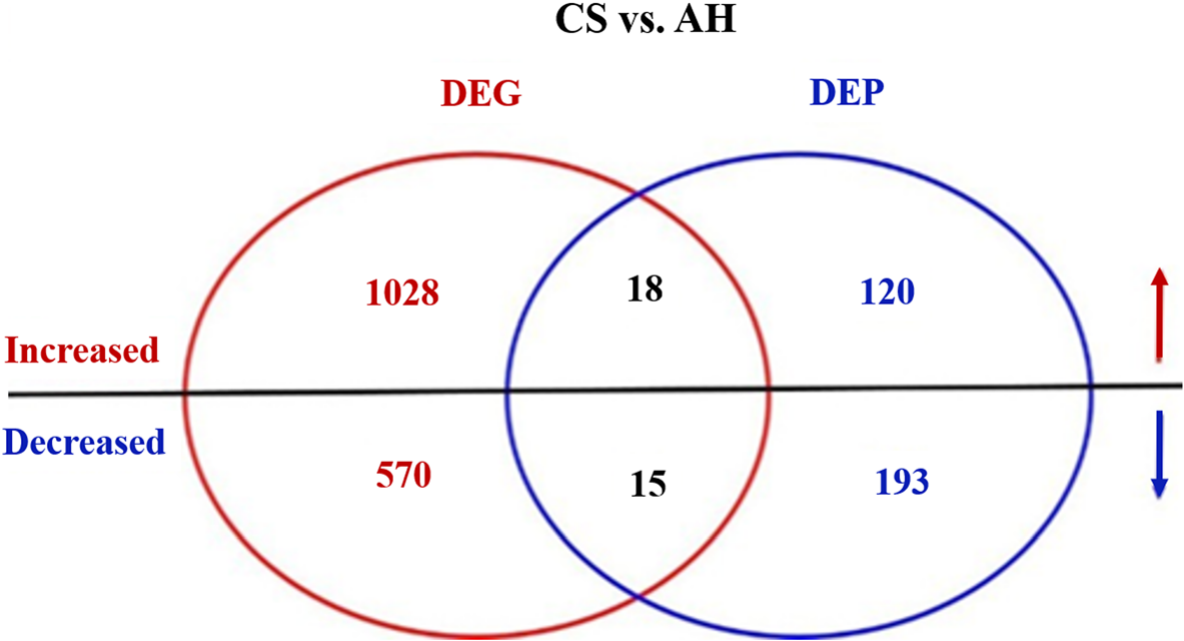
The venn diagram of the differentially expressed genes (DEGs) and proteins (DEPs) in the mammary gland of cows fed either corn stover (CS) or alfalfa hay (AH). The cut-off of differential expression of mRNA is set at 1.5-fold change and *p* < 0.05, whereas the cutoff of differential expression of protein is set at 1.2-fold and *p* < 0.05

Figure 1 shows the workflow of our integrative RNA-seq transcriptomic and iTRAQ-based proteomic experiments. In the transcriptomic analysis, 78,138,798 and 90,522,588 raw/clean reads were detected in the CS and AH groups, respectively (Additional file 2: Table S2), and in proteomic analysis, 62,367 unique spectra were strictly matched to 24,606 unique peptides and further mapped to 3744 unique proteins (Additional file 2: Table S3). With a cutoff of 1.5-fold change and a *p*-value < 0.05, a total of 1631 differentially expressed genes (DEGs) were identified between CS and AH groups in transcriptomic analysis, among which 1046 DEGs were up-regulated and 585 were down-regulated (Fig. 2 and Additional file 3: Table S4). Using a threshold of 1.2-fold change and *p* < 0.05, 346 differentially expressed proteins (DEPs; 138 up-regulated and 208 down-regulated) were detected between CS and AH groups in proteomic analysis (Fig. 2 and Additional file 3: Table S5). By comparing the RNA-seq data with the proteomic data (Fig. 2 and Additional file 3: Table S6), 40 genes displayed differential expression at both mRNA and protein levels, of which 18 and 15 genes were consistently up- or down-regulated, respectively, whereas the remaining 7 genes had inconsistent expression in mRNA and protein levels, which may result from post-translational modifications.

### The gene ontology (GO) analysis of DEGs and DEPs

**Fig. 3:**
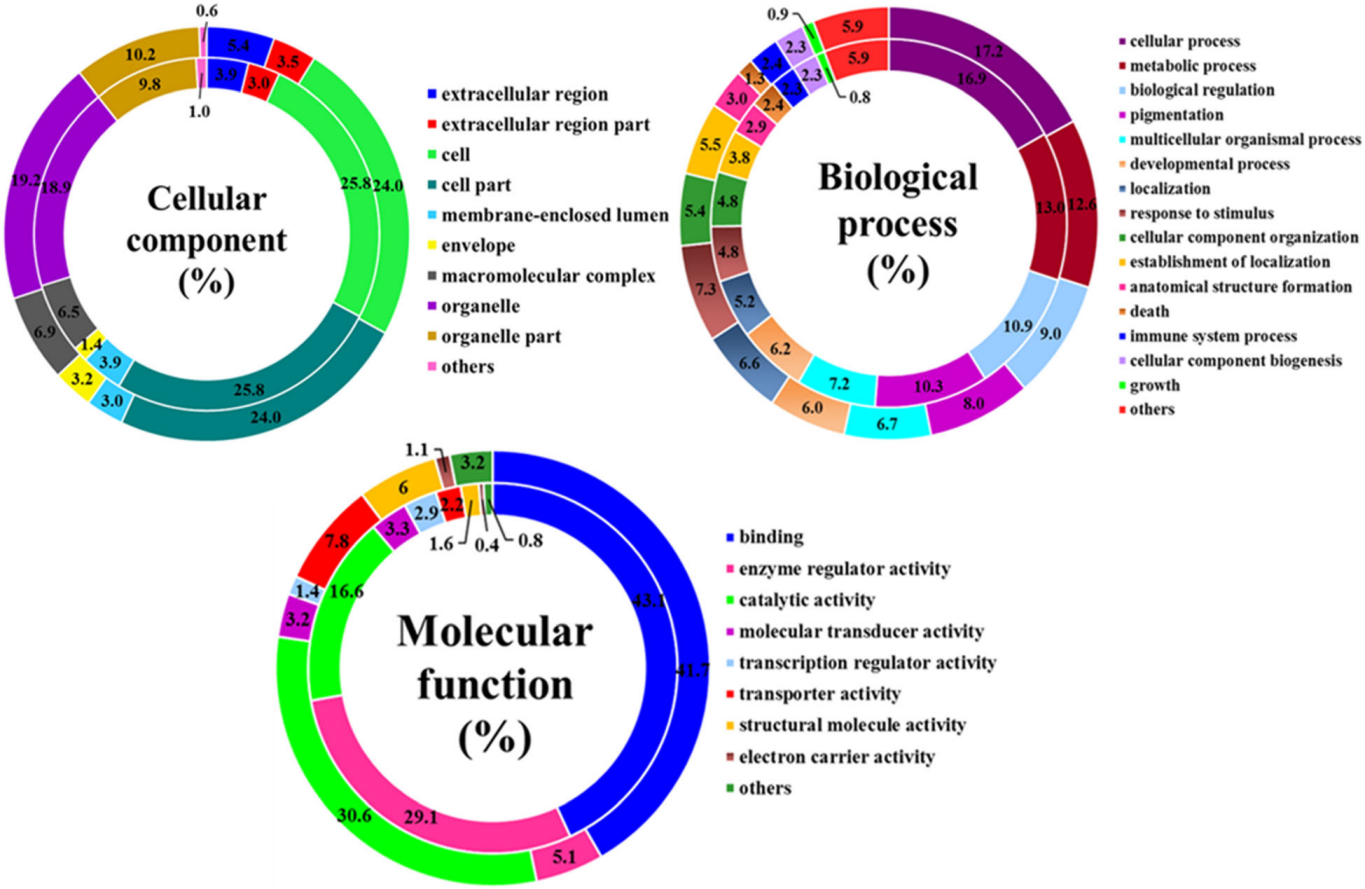
Gene ontology (GO) categories assigned to the differentially expressed genes (DEGs, inner cycle) and proteins (DEPs, outer cycle) in the mammary gland of cows fed either corn stover (CS) or alfalfa hay (AH). The differentially expressed genes were classified into cellular component, biological process, and molecular function by WEGO (Web Gene Ontology Annotation Plot) according to the GO terms

The GO analysis of all the DEGs and DEPs in cows fed CS versus AH is shown in Fig. 3. In the cellular component category, most of the DEGs and DEPs were mainly assigned to cell (25.8% genes and 24% proteins), cell part (25.8% genes and 24% proteins), organelle (18.9% genes and 19.2% proteins) and organelle part (9.8% genes and 10.2% proteins). Notably, a small number of the DEGs and DEPs were located in the extracellular region (3.9% genes and 5.4% proteins), membrane-enclosed lumen (3.9% genes and 3.0% proteins) and macromolecular complexes (6.5% genes and 6.9% proteins). In the biological process category, a large number of DEGs and DEPs were involved in cellular process (16.9% genes and 17.2% proteins), metabolic process (13.0% genes and 12.6% proteins) and biological regulation (10.9% genes and 9.0% proteins). Noticeably, some DEGs and DEPs were assigned to response to stimulus (4.8% genes and 7.3% proteins), immune system process (2.3% genes and 2.4% proteins), cellular component biogenesis (2.3% genes and 2.3% proteins) and cell growth (0.8% genes and 0.9% proteins). For the molecular function category, the GO terms including binding (43.1% genes and 41.7% proteins), enzyme regulator activity (29.1% genes and 5.1% proteins) and catalytic activity (16.6% genes and 30.6% proteins) were the predominant functions of the DEGs and DEPs, and a relatively low proportion of DEGs and DEPs were associated with various biological activities such as molecular transducer (3.3% genes and 3.2% proteins), transcription regulator (2.9% genes and 1.4% proteins), transporter (2.2% genes and 7.8% proteins), structural molecule (1.6% genes and 6.0% proteins) and electron carrier (0.4% genes and 1.1% proteins). In general, the DEGs and DEPs displayed similar GO annotation patterns.

**Fig. 4:**
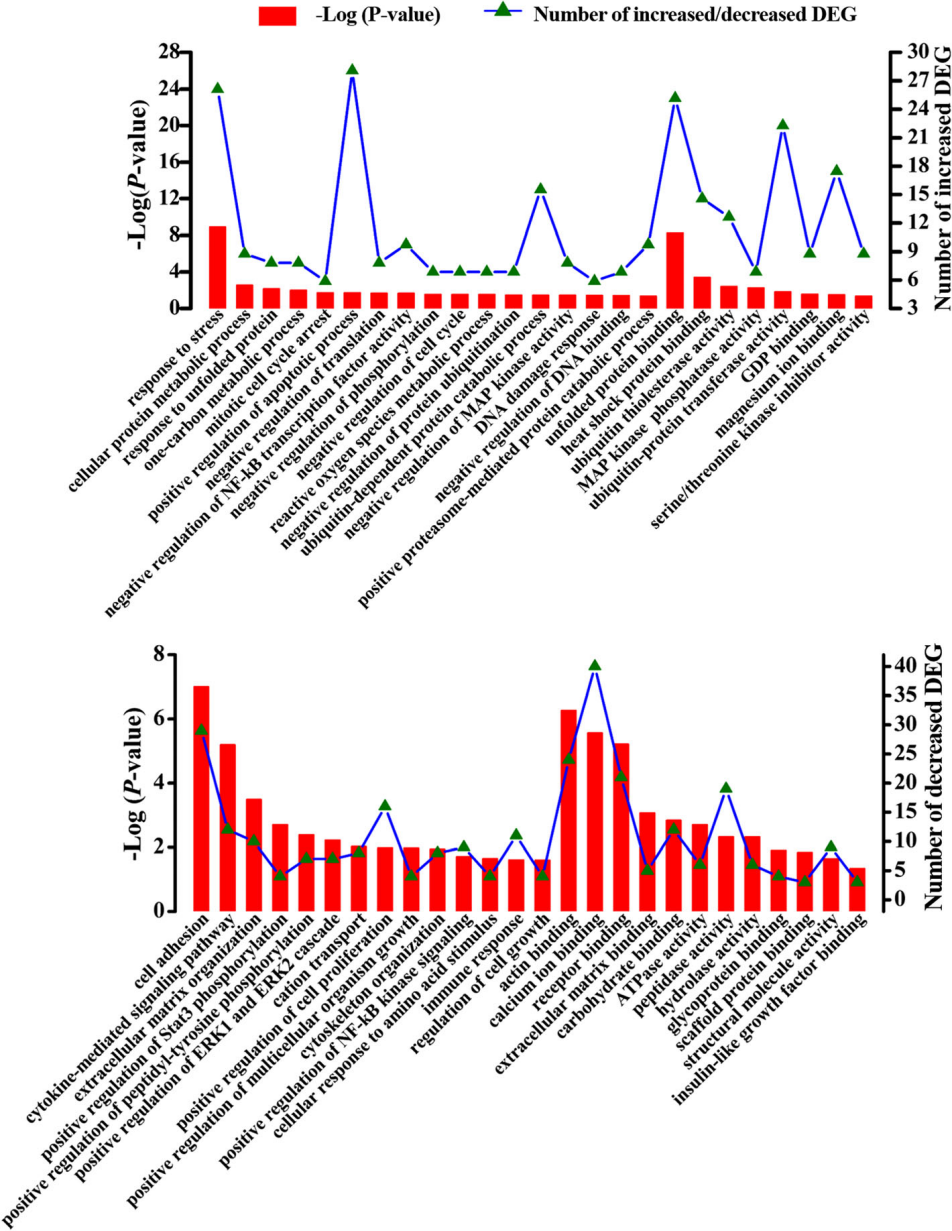
Functional characterization of the increased or decreased transcripts in the mammary gland of cows fed alfalfa hay (AH) vs. corn stover (CS) by gene ontology analysis. DEG indicates differentially expressed genes. The x-axis shows the functional categories of the increased or decreased genes, the left y-axis shows the value of –Log (*p*-value) and the right y-axis shows the number of increased/decreased genes

We also performed further functional analysis of the up- and down-regulated genes and proteins using the UniProt knowledgebase and GO database. The increased genes (Fig. 4) in the CS group were significantly and abundantly enriched for genes involved in the positive regulation of apoptosis, unfolded protein binding, negative regulation of protein ubiquitination and response to stress. In addition, we found that some genes with increased abundance were enriched in the negative regulations of translation, DNA binding, NF-κB transcription factor activity, phosphorylation, cell cycle and in proteasome-mediated/ubiquitin-dependent protein catabolic processes. Notably, nine genes with up-regulated expression were enriched in the GO term GDP binding. In contrast, the decreased genes (Fig. 4) were principally enriched in cell adhesion, positive regulation of cell proliferation, binding activities of actin/receptor/carbohydrate, structural molecule activity and protein binding (295 decreased genes enriched). Additionally, some down-regulated genes were related to the positive regulation of several transcriptional factors (Stat3 phosphorylation and NF-κB signaling) and cell-growth associated processes (cell proliferation, multicellular organism growth, and cell growth). Importantly, a small number of decreased genes were associated with the cytoskeleton/extracellular matrix and cytokine-mediated signaling pathway. Notably, four decreased genes were significantly enriched in the GO term “cellular response to amino acid stimulus”. Furthermore, the GO term “ATPase activity” was significantly enriched in six down-regulated genes.

**Fig. 5:**
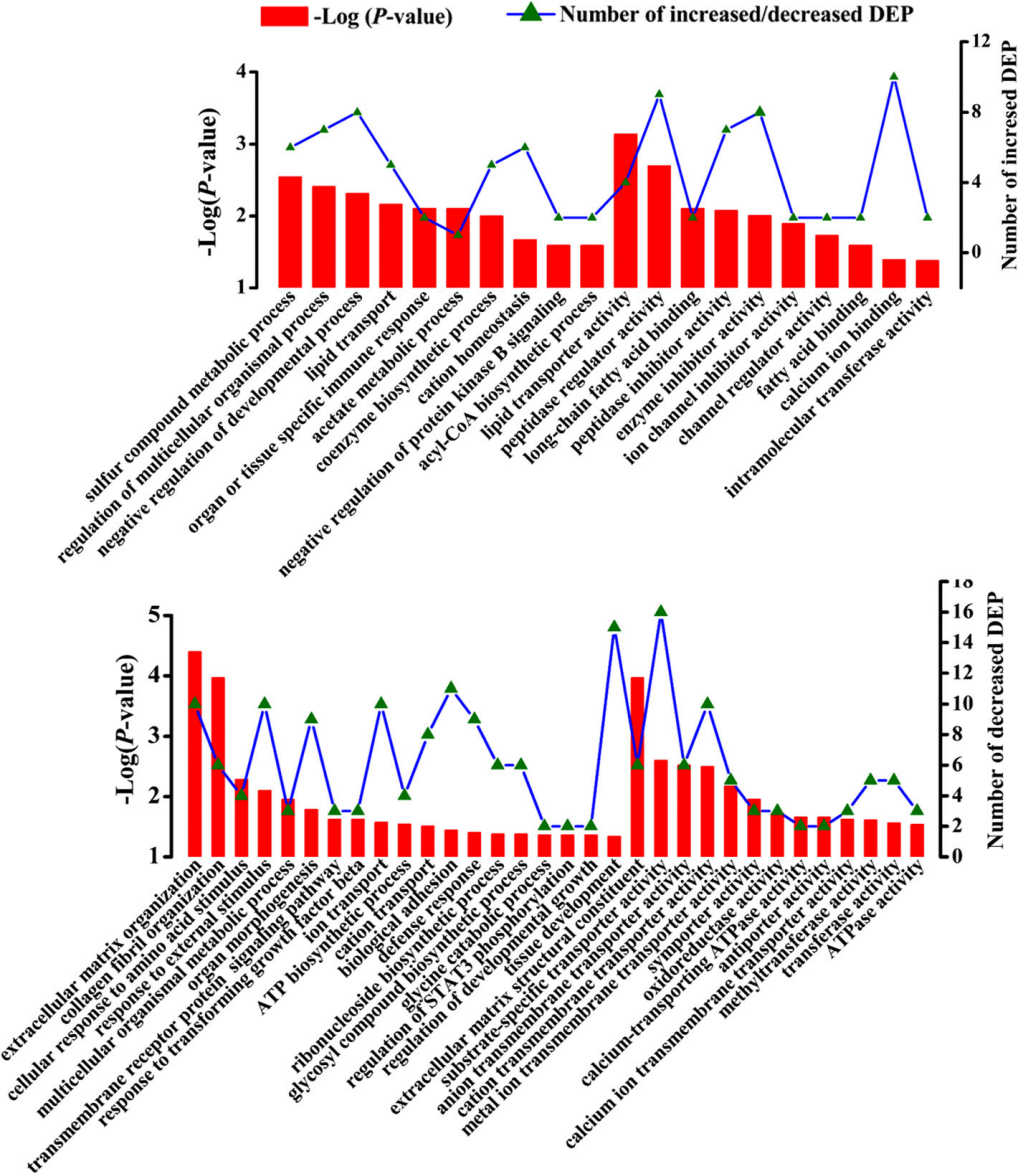
Functional characterization of the increased or decreased proteins in the mammary gland of cows fed alfalfa hay (AH) vs. corn stover (CS) by gene ontology analysis. DEP indicates differentially expressed proteins. The x-axis shows the functional categories of increased or decreased proteins, the left y-axis shows the value of –Log (*p*-value) and the right y-axis shows the number of increased/decreased proteins

The up-regulated proteins (Fig. 5) were primarily enriched in negative regulation of multicellular organismal and developmental processes. In addition, we observed a small proportion of increased proteins enriched in the GO terms related to lipid translocation including lipid transport and long-chain fatty acid binding, and in GO terms involved in cation homeostasis and ion channel inhibitor activity. Specifically, eight up-regulated proteins were enriched in enzyme inhibitor activity, which may suggest a general reduction of the enzyme-mediated metabolism. The GO annotation analysis of the down-regulated proteins is shown in Fig. 5. Notably, the most abundant GO term was extracellular matrix organization enriched in ten down-regulated proteins. In addition, the body defense-related GO terms, such as response to external stimulus, cellular response to amino acid stimulus, and defense response, were significantly enriched with some decreased proteins. Some proteins of down-regulation were involved in tissue development, organ morphogenesis, biological adhesion and response to transforming growth factor beta. Importantly, 16 down-regulated proteins were involved in substrate-specific transporter activity. Furthermore, six decreased proteins were enriched in glycosyl-compound biosynthetic process and three decreased proteins were related to ATPase activity.

### KEGG pathway analysis of DEGs and DEPs

Table 1 shows the KEGG pathway enrichment analysis of DEGs. In the analysis, a total of 7 decreased DEGs were significantly enriched in the pathway of protein digestion and absorption (*p* = 0.0269). In contrast, 10 increased DEGs were significantly enriched in protein processing in endoplasmic reticulum (*p* = 0.045). Additionally, we found that 4, 11, and 7 increased genes were significantly enriched in protein degradation-related processes ubiquitin mediated proteolysis, spliceosome and proteasome, respectively. In particular, the significant pathways (*p* ≤ 0.05) enriched by some decreased DEGs were related to protein synthesis (such as ribosome biogenesis in eukaryotes, aminoacyl-tRNA biosynthesis and lysosome). Also, a small number of DEGs were significantly enriched in the pathways of mTOR signaling pathway, p53 signaling pathway, and two cell-growth-associated signaling pathways (the TGF-beta signaling and Wnt signaling).

**Table 1:** The KEGG pathway enrichment by up−/down-regulated genes in the mammary gland of cows fed corn stover (CS) vs. alfalfa hay (AH)

KEGG ID	Pathway Name	*P*-value of Fisher’ exact Test	No. of Increased Genes	ID of Increased Genes	Gene Symbol of Increased Genes	No. of Decreased Genes	ID of Decreased Genes	Gene Symbol of Decreased Genes
ko04974	Protein digestion and absorption	0.0269	0			7	XLOC_022008;XLOC_001311; XLOC_000568;XLOC_027248; XLOC_000569;XLOC_017584; XLOC_020771	SLC38A2; SLC7A8; BT.23508; COL12A1; COL6A2; COL17A1; COL6A3
ko04141	Protein processing in endoplasmic reticulum	0.0450	10	XLOC_000859;XLOC_003872;XLOC_006584;XLOC_009297;XLOC_020319;XLOC_022100;XLOC_024455;XLOC_025681;XLOC_026233;XLOC_027001	HSPH1;DNAJB1;DNAJA1;DDIT3;CRYAB;SAR1B;BT.59327;MAN1A2;SEC63;DNAJB11	0		
ko04120	Ubiquitin mediated proteolysis	0.0277	4	XLOC_024865;XLOC_018298;XLOC_021649;XLOC_025981	UBE2B;HERC4;UBE2H;BT.19212	1	XLOC_025357	KEAP1
ko0970	Aminoacyl-tRNA biosynthesis	0.0312				1	XLOC_025065	RARS
ko03010	Ribosome	0.0201				1	XLOC_028013	RPS23
ko03008	Ribosome biogenesis in eukaryotes	0.0219	2	XLOC_001726; XLOC_010292	FCF1;UTP6	3	XLOC_025000;XLOC_023340;XLOC_026685	TCOF1;RRP7A;NOL6
ko03040	Spliceosome	0.0388	11	XLOC_000309;XLOC_004289;XLOC_007964;XLOC_012182;XLOC_012566;XLOC_017604;XLOC_019531;XLOC_021421;XLOC_023325;XLOC_025841;XLOC_028252	SNRPB2;TRA2B;SMNDC1;TRA2A;BCAS2;SF3B1;PHF5A;BT.59135;SLU7;BT.91058;BT.91058;PLRG1	1	XLOC_012196	SFRS4
ko03050	Proteasome	0.0142	7	XLOC_001624;XLOC_001843;XLOC_003577;XLOC_004136;XLOC_012473;XLOC_020934;XLOC_021072	BT.22570;PSMD14;PSMA3;BT.56882;PSMA2;POMP;PSMC2			
ko04142	Lysosome	0.0394	0			5	XLOC_001832;XLOC_006153;XLOC_010442;XLOC_012190;XLOC_013979	BT.35140; LAPTM5; ARSB; CD68; CTSH
ko04150	mTOR signaling pathway	0.0300	1	XLOC_018127	DDIT4	1	XLOC_013054;	RICTOR
ko04350	TGF-beta signaling pathway	0.0190	2	XLOC_003540;XLOC_018178	ID2;BT.48514	3	XLOC_004021;XLOC_004591;XLOC_013257	FST;ID1;TFDP1
ko04115	p53 signaling pathway	0.0247	5	XLOC_007111;XLOC_012771;XLOC_025054;XLOC_026769;XLOC_026996	GADD45G;SESN1;BT.36413;SESN2;CCNG1	2	XLOC_021046;XLOC_018555	IGFBP3;BT.33239
ko04310	Wnt signaling pathway	0.0458	1	XLOC_007394	CACYBP	1	XLOC_007966	SFRP2
ko03050	Proteasome	0.0142	7	XLOC_001624;XLOC_001843;XLOC_003577;XLOC_004136;XLOC_012473;XLOC_020934;XLOC_021072	BT.22570;PSMD14;PSMA3;BT.56882;PSMA2;POMP;PSMC2			

The KEGG pathway enrichment analysis of the DEPs is shown in Table 2, and a total of 29 KEGG pathways were significantly enriched. Importantly, 7 decreased DEPs were significantly enriched (*p* = 0.0101) in the “protein digestion and absorption” pathway, while 7 increased DEPs were significantly enriched (*p* = 0.037) in the “protein processing in endoplasmic and reticulum” pathway. Several DEPs were involved in pathways associated with energy metabolism― glycolysis/gluconeogenesis (*p* = 0.0302), citrate cycle (*p* = 0.0399), pentose phosphate pathway (*p* = 0.0352), and PI3K-Akt signaling (*p* = 0.0375). Additionally, the KEGG pathways enriched by a small number of DEPs were related to the protein synthesis/processing (including ribosome, *p* = 0.0069; aminoacyl-tRNA biosynthesis, *p* = 0.0236; mTOR signaling pathway, *p* = 0.035; lysosome, *p* = 0.0350; protein processing in endoplasmic reticulum, *p* = 0.0370; and spliceosome, *p* = 0.0232) and the metabolisms of several amino acids. Noticeably, we also found that a certain of proteins were significantly enriched in two cell-growth-signaling pathways (Wnt and TGF-beta signaling pathways; *p* = 0.0375 and 0.0272, respectively).

**Table 2:** The KEGG pathway enrichment by up−/down-regulated proteins in the mammary gland of cows fed corn stover (CS) vs. alfalfa hay (AH)

KEGG ID	Pathway Name	*P*-value of Fisher’ exact Test	No. of Increased Transcripts	ID of Increased Proteins	Gene Symbol of Increased Proteins	No. of Decreased Proteins	ID of Decreased Proteins	Gene Symbol of Decreased Proteins
ko03040	Spliceosome	0.0232	4	IPI00687479;IPI00715218; IPI00702381; IPI00687395	SNRNP40;LSM3; SNRPB;PRPF8	6	IPI00690232;IPI00699558;IPI00687560;IPI00687015;IPI00717302; IPI00688521	MAGOHB;SNRPD3;PCBP1;SNRPD2;SF3B4;BUD31
ko04141	Protein processing in endoplasmic reticulum	0.0370	7	IPI00702891;IPI00699038; IPI00693007; IPI00699107;IPI00691963; IPI00696616;IPI00688461	ERP29; TXNDC5; DNAJC3;DNAJB11; CALR; SSR2; DNAJB1	1	IPI00692963	SEC23
ko04974	Protein digestion and absorption	0.0101	0			7	IPI00707857;IPI00708244;IPI00711933;IPI00712524;IPI00731432;IPI00826022;IPI00905045	COL4A2; COL3A1; COL5A2; COL1A1; COL1A2;COL11A1; COL18A1
ko04142	Lysosome	0.0314	2	IPI00711862; IPI00706203	NPC2; HEXB	4	IPI00697314;IPI00699372;IPI00717554;IPI00716195	CTSC; ATP6V0A1; NAGLU; ATP6V0D1
ko03010	Ribosome	0.0069	0			6	IPI00695732;IPI00699146;IPI00707431;IPI00713536;IPI00714445;IPI00715091	RPS2; RPS16; RPS20; RPS19; RPS12; RPS27
ko00260	Glycine, serine and threonine metabolism	0.0090	1	IPI00698589	PGAM1	3	IPI00698059;IPI00707303;IPI00715285	SARDH; DMGDH; MAOA
ko04150	mTOR signaling pathway	0.0350	1	IPI00700182	EIF4B	2	IPI00903663; IPI00732002	IKBKB; MAPK3
ko03008	Ribosome biogenesis in eukaryotes	0.0374	2	IPI00705941;IPI00708018	REXO2; RAN	1	IPI00852474	NAT10
ko00280	Valine, leucine and isoleucine degradation	0.0468	3	IPI00711918;IPI00717256;IPI00968674	DBT; ACAT1; HMGCS1			
ko00380	Tryptophan metabolism	0.0381	1	IPI00711918	ACAT1	1	IPI00698059	MAOA
ko00330	Arginine and proline metabolism	0.0370	0			2	IPI00698059;IPI00838420	P4HA2; MAOA
ko00970	Aminoacyl-tRNA biosynthesis	0.0236	0			2	IPI00689365;IPI00703906	TARS2; AARS2
ko00360	Phenylalanine metabolism	0.0473	0			1	IPI00698059	MAOA
ko00340	Histidine metabolism	0.0461	0			1	IPI00698059	MAOA
ko00350	Tyrosine metabolism	0.0365	0			1	IPI00698059	MAOA
ko00270	Cysteine and methionine metabolism	0.0432	1	IPI00694739	APIP			
ko00010	Glycolysis / Gluconeogenesis	0.0302	3	IPI00696912; IPI00698589; IPI00712164	ACSS1; PGAM1; GALM	2	IPI00687211;IPI00715799	HK1; GAPDHS
ko00190	Oxidative phosphorylation	0.0287	0			5	IPI00697768;IPI00699372;IPI00712252;IPI00716163;IPI00716195	ATPsynGL;ATP6V0A1; ATP5H;NDUFC1; ATP6V0D1
ko00020	Citrate cycle (TCA cycle)	0.0399	2	IPI00702781;IPI00708438	IDH1; SUCLG1	1	IPI00714468	IDH2
ko00640	Propanoate metabolism	0.0423	3	IPI00696912;IPI00708438;IPI00711918	ACSS1; ACAT1; SUCLG1			
ko00030	Pentose phosphate pathway	0.0352	2	IPI00728589;IPI00904104	TKT; RBKS			
ko04146	Peroxisome	0.0169	4	IPI00686601;IPI00702781;IPI00704382;IPI00714468	SOD2; SCP2; IDH1; ECH1	1	IPI00714468	IDH2
ko03320	PPAR signaling pathway	0.0263	4	IPI00686601;IPI00699355;IPI00715548;IPI00839653	SCP2; FABP4; APOA1; PPARD			
ko04975	Fat digestion and absorption	0.0370	2	IPI00695965;IPI00715548	APOA4; APOA1	1	IPI00710056	APOB
ko04540	Gap junction	0.0352	0			2	IPI00695917;IPI00732002	MAPK3; GNAS
ko04210	Apoptosis	0.0261	1	IPI00704835	DFFA	2	IPI00709124; IPI00903663	ENDOG; IKBKB
ko04151	PI3K-Akt signaling pathway	0.0301	1	IPI00700182	EIF4B	9	IPI00697595;IPI00707857;IPI00708244;IPI00712524;IPI00731432;IPI00732002;IPI00826022;IPI00903663;IPI00905045	COL4A2; IKBKB; COL3A1; COL5A2; COL1A1; COL1A2; COL11A1; MAPK3; ITGA1
ko04310	Wnt signaling pathway	0.0375	1	IPI00708311	CACYBP	1	IPI00699355	PPARD
ko04350	TGF-beta signaling pathway	0.0272	0			1	IPI00732002	MAPK3

### Functional analysis of the common DEGs and DEPs

The 40 common expressed genes at mRNA and protein levels were further analyzed by the GO and KEGG pathway (Additional file 4: Table S7). Importantly, 3 increased genes at mRNA and protein levels (DnaJ homolog subfamily B members—*DNAJB11*, *DNAJB1*, and *DNAJC12*) were related to protein unfolding and involved in the pathway of “protein processing in endoplasmic reticulum”. Also, two common genes of up-regulation (dihydrolipoamide branched chain transacylase, *DBT* and hydroxymethylglutaryl-CoA synthase, *HMGCS1*) were involved in the process of “valine, leucine and isoleucine degradation”. Noticeably, the decreased genes *IDH2* and ATPase 2 (*ATP2B4*) at mRNA and protein levels were involved in TCA cycle and ATP synthesis, respectively; in contrast, 4 common genes of up-regulated expression [*HMGCS1*, *SCP2*, *DBT* and isopentenyl-diphosphate delta-isomerase 1 (*IDI1*))] were all associated with fatty-acyl-CoA metabolic process. In particular, the commonly decreased gene dimethylglycine dehydrogenase (*DMGDH*) was associated with “glycine, serine and threonine metabolism”. In addition, several commonly expressed genes of down-regulation [such as erythrocyte membrane protein (*EPB41L3*), odorant-binding protein (*MGC151921*), *DMGDH*, VPS18 protein (*VPS18*), and the uncharacterized protein *ORAI1*] were involved in the processes of “protein localization” and “transport”. Intriguingly, two up-regulated genes *DHFR* and *KRT15* at mRNA and protein levels were related to one carbon metabolism and *staphylococcus aureus* infection, respectively. Additionally, the 3 collagens (*COL1A1*, *COL1A2* and *COL4A2*) and mitogen-activated protein kinase 3 (*MAPK3)* down-regulated at both mRNA and protein levels were involved in the protein synthesis-related pathways of “protein digestion and absorption” and “ECM-receptor interaction”.

### Verification of DEGs by qRT-PCR and DEPs by western blot analysis

**Fig. 6:**
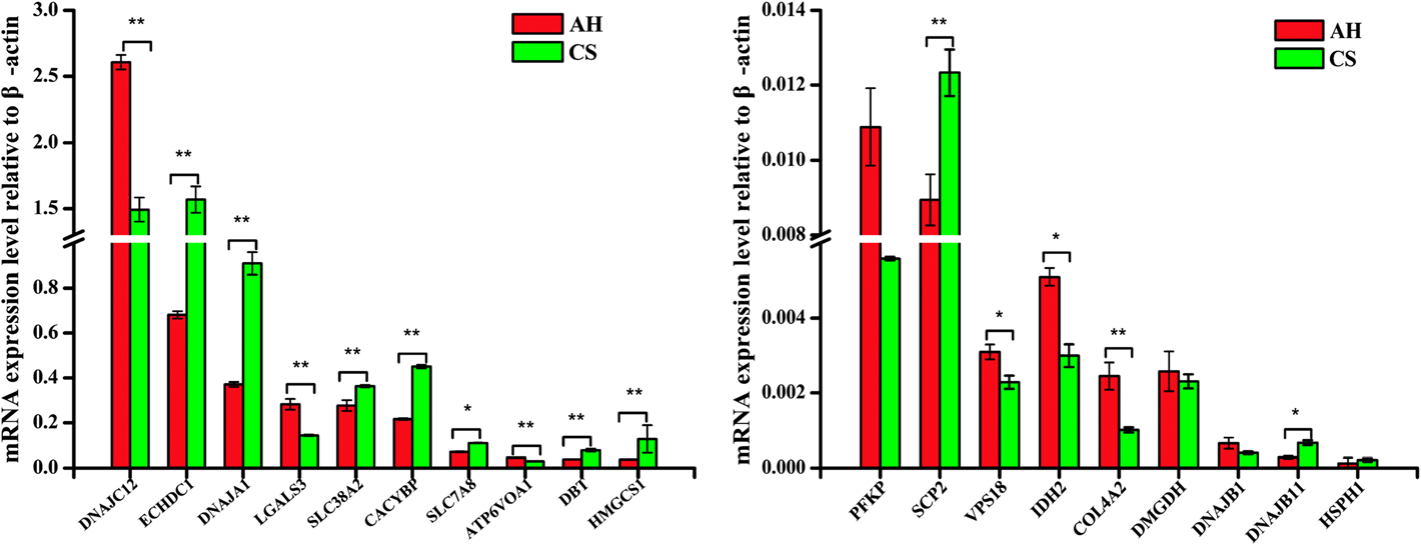
Real time PCR analysis of mRNA expression changes of genes involved in mammary metabolism of cows fed corn stover (CS) and alfalfa hay (AH). Relative mRNA expression levels were normalized by the levels of β-actin. Error bars represent the standard deviation. ** and * indicate that the difference in gene expression between CS and AH groups reached *p* < 0.01, and 0.01 < *p* < 0.05, respectively

**Fig. 7:**
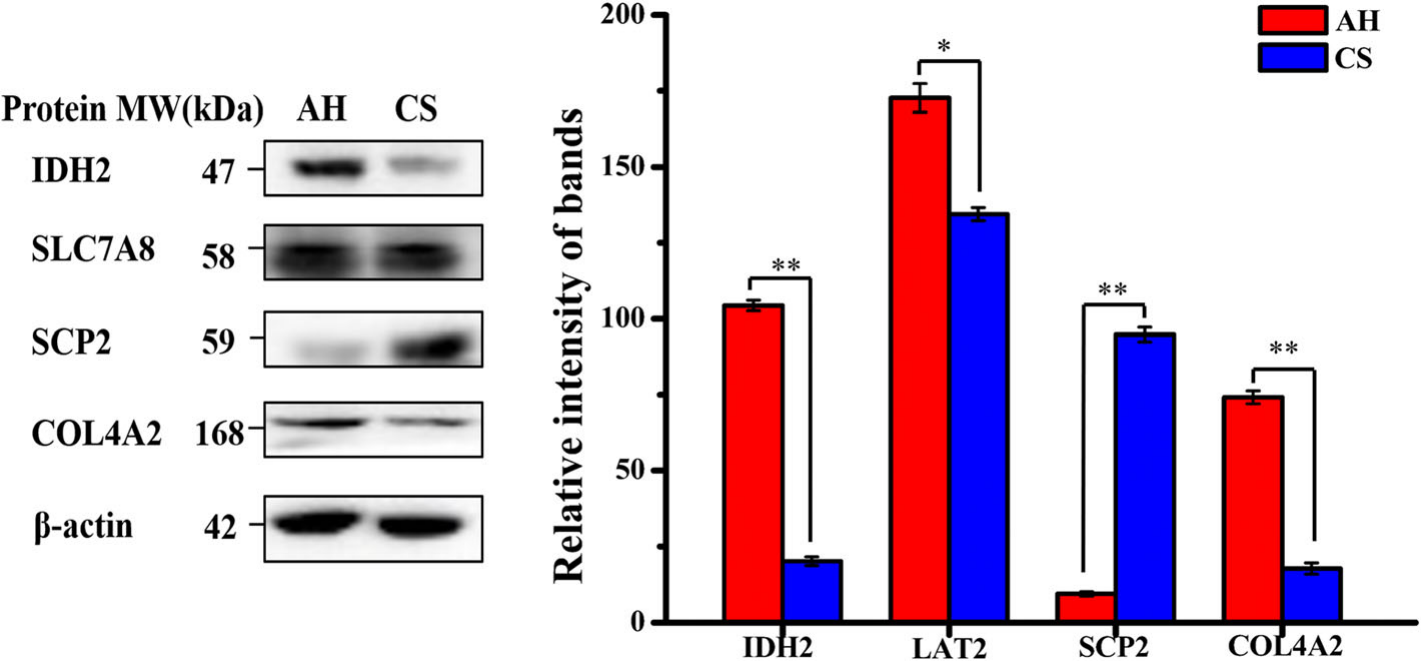
Western blot analysis of expression of IDH2, SLC7A8, SCP2, and COL4A2 proteins in the mammary gland of cows fed corn stover (CS) and alfalfa hay (AH). β-Actin was used as a sample loading control. ** and * indicate *p* < 0.01 and 0.01 < *p* < 0.05, respectively. IDH2: Isocitrate dehydrogenase 2; SLC7A8: also referred as LAT2, L type amino acid transporter 2; SCP2: sterol carrier protein 2; COL4A2: collagen type IV alpha 2

Nineteen genes involved in energy metabolism, transcription/translation, protein processing/transport, protein degradation, amino acid metabolism/ transport, fatty acid oxidation, and mammary gland growth and development were selected for qRT-PCR analysis (Additional file 5: Table S8). Among these genes (Fig. 6), expression levels of 15 genes were significantly altered between CS- and AH- groups, and the abundance of 11 of the 15 gene abundance were consistent with expression patterns measured by RNA-seq. Four proteins, including *IDH2*, *SLC7A8*, *SCP2*, and *COL4A2* were selected for Western blot analysis (Fig. 7 and Additional file 5: Table S8). Western blot analysis showed that the protein levels of *IDH2*, *SLC7A8*, and *COL4A2* in the mammary glands of cows fed the CS-based diet were lower than those cows fed the AH diet, whereas the level of *SCP2* was higher. All the immunoblot results were consistent with the findings from proteomic analysis.

### Relationship between the DEGs/DEPs and mammary metabolism

**Fig. 8:**
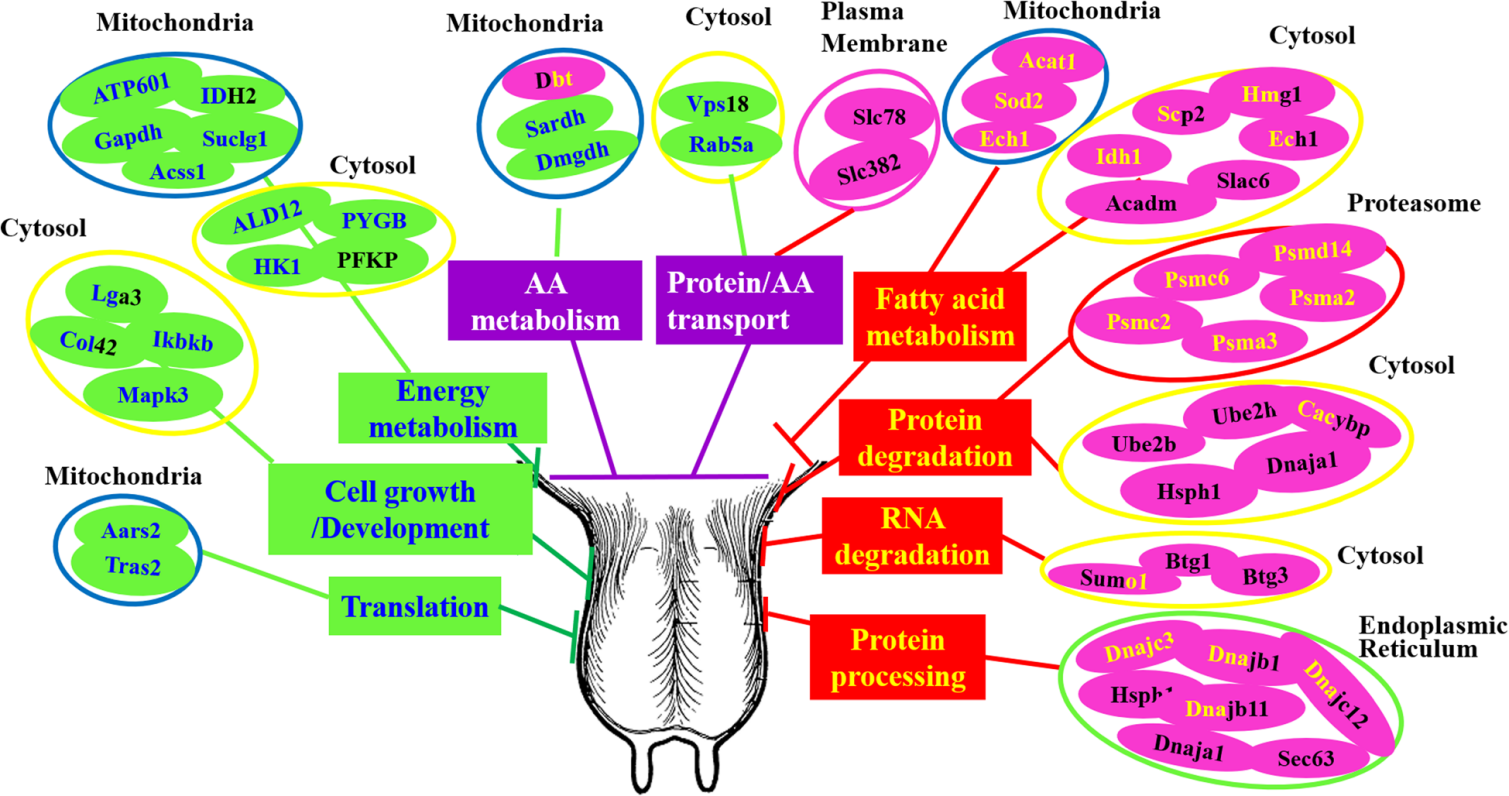
An overview of possible biological changes that might contribute to low milk production in cows fed corn stover-based diet vs. cows fed alfalfa hay-based diet. The color coding for the individual genes is as follows: black letters and pink background represents the up-regulated genes, yellow letters and pink background represents increased proteins, black letters and green background represents the down-regulated genes, and blue letters and green background represents decreased proteins. The half blue/half black letters with green background represents genes that were both down-regulated at mRNA and protein levels. The half black/half yellow letters with pink background represent the genes that were up-regulated at both protein and gene levels. The full name of each protein is listed in Additional file 6: Table S9

A comprehensive view of the molecular mechanisms underlying milk production was summarized based on the proteomic and transcriptomic data collected (Fig. 8 and Additional file 6: Table S9). The regulatory subsections proposed include: energy metabolism, amino acid (AA) /fatty acid metabolism, protein degradation, protein synthesis, protein processing, AA/protein transport, and cell growth and development. Each of these regulatory functions is involved in the regulation of mammary metabolism, and the collective analysis shows how the mammary gland adapts to the low nutrient availability of the CS ration compared with the higher nutrient availability on the AH ration. Despite that the gene- and protein-level responses are not always consistent, the relatively higher degree of agreement between analyses of DEGs and DEPs at the functional level suggests that transcriptomic analysis of the mammary gland might be sufficient to characterize tissue functional responses to altered states but might not always be reflective of shifts in specific proteins.

## Discussion

Roughage is one of the most important components in ruminant diets, the quality of which has a large impact on milk performance in dairy cows [25]. Our previous study showed that compared to cows fed AH as the forage source, cows fed CS had lower yields of milk (kg/d; 23.5 vs. 19.4), milk fat (kg/d; 0.98 vs. 0.82), milk protein (kg/d; 0.77 vs. 0.62) and lactose (kg/d; 1.15 vs. 0.94) (*P* < 0.01) [6]. In addition, as a major crop byproduct, a large quantity of corn stover is burned by humans, and this inefficient utilization of crop residues subsequently produces heavy environmental pollution. Therefore, it is urgent for us to determine strategies to improve the utilization of low-quality forage (CS) in the dairy industry and reduce damage to the environment caused by burning CS. However, little is known about the effects of roughage consumption on the molecular mechanisms in milk production in the mammary gland. In this study, we detected 1046 up-regulated and 585 down-regulated genes by RNA-seq transcriptomics as well as 138 increased and 208 decreased proteins by iTRAQ proteomics in the mammary gland of dairy cows fed CS- versus AH-based diets. There were only 33 genes with consistent expression patterns measured by both transcriptomic and proteomic analyses, suggesting that the post-transcriptional regulation may play an important role in gene expression. The relatively limited overlap of proteins and mRNAs was also seen in other studies in human [14], mouse [11], fish [26] and bacteria [27].

The GO term annotation can help to characterize physiological and functional changes associated with the changes in mRNA and protein expression in cells and tissues [28]. In up-regulated genes in CS-fed cows versus AH-fed cows, the GO term “ubiquitin-dependent/proteasome-mediated protein catabolic process” was significantly enriched, which may indicate enhanced mammary protein catabolism in cows fed CS. Enrichment in the GO terms of negative regulation of translation, NF-κB transcription factor activity, and DNA binding activity suggested inhibited protein synthesis in the mammary gland, consistent with the reduced milk protein yield in CS-fed cows [6]. In addition, several proteins that were up-regulated in CS-fed cows were enriched in the GO term “enzyme inhibitor activity”, which is also consistent with an overall reduced mammary metabolism in these cows. Furthermore, some increased proteins were involved in positive regulation of apoptosis and negative regulations of multicellular organismal processes and developmental processes, in line with the reduced cell growth in the CS group as shown in our previous microRNAome analysis [8]. Interestingly, the fact that the GO terms of lipid transport and long-fatty acid binding were enriched in the up-regulated proteins in CS-fed cows supported the idea that the mammary gland of these cows may take up more fatty acids from the blood for mammary metabolism as these cows had a lower acetate supply to the mammary gland than AH-fed cows [6].

Consistently, the genes that were expressed lower in CS-fed cows were enriched for the GO terms of cell adhesion, positive regulation of cell proliferation, and multicellular organism growth, which also indicated the attenuated cell growth in the mammary gland of CS-fed animals. In addition, down-regulation of genes associated with positive regulation of Stat3 phosphorylation and NF-κB signaling pathway in CS-fed cows was consistent with the reduced protein synthesis in these animals. Meanwhile, the lower expression levels of the proteins involved in biological adhesion, extracellular matrix organization, tissue development, and organ morphogenesis were consistent with possible reduced mammary cell growth and mammary tissue development in CS-fed cows. Furthermore, the proteins that were lower in abundance were enriched in the GO term “response to transforming growth factor beta”, indicating that TGF-β may be a major signaling molecule in the regulation of mammary cell growth in cows fed CS. Collectively, GO analysis indicated that the reduced cell growth and metabolism, attenuated protein synthesis, and enhanced protein degradation may play major roles leading to the low milk production in CS-fed dairy cows.

The KEGG pathway analysis has also been widely used for systematic understanding of the gene functions in cells or organisms from large-scale molecular data sets [29, 30]. In this study, the up-regulated genes were enriched in ubiquitin mediated proteolysis, and the genes with lower abundance were enriched in ribosome (the main machinery for protein synthesis), suggesting that these pathways may play an integrative role in lowering milk protein production in the mammary gland of CS-fed cows [6]. Additionally, some down-regulated proteins from CS-fed cows were enriched in the mTOR signaling pathway and metabolisms of several amino acids, including glycine, serine, threonine, arginine, proline, phenylalanine, histidine, and tyrosine, which is also consistent with the lower milk protein in the CS group [6, 8]. In particular, three down-regulated proteins in CS-fed cows were associated with valine, leucine and isoleucine degradation, indicating a reduction of branched-amino acids production in milk [31]. Moreover, the pathways enriched among differentially expressed proteins included the Wnt and TGF-β signaling pathways. These pathways are known to play roles in regulating mammary growth and differentiation [32–34].

Milk production is a highly energy-dependent process and requires sufficient ATP [35]. In most eukaryotes, oxidative phosphorylation in the mitochondria involving a series of ATPases is the prime metabolic pathway to generate energy [36]. In the study, we observed that the expression of several ATPase components (including *ATPsynGL*, *ATP6V0A1*, *ATP5H* and *ATP6V0D1*) and one mitochondrial enzyme NADH dehydrogenase (ubiquinone) 1 subunit C1 (*NDUFC1*) involved in oxidative phosphorylation were lower in CS-fed cows. The lower levels of these enzymes may potentially contribute to a lower ATP production in the mammary tissue of these animals. For high-yield dairy cows, particularly during negative energy balance, ketone bodies (included acetoacetate, β-hydroxy-butyrate, and acetone) are produced in the liver but utilized in other tissues of the body (including the mammary gland) as an energy source [37]. Acetyl-CoA acetyltransferase (*ACAT1*) plays a major role in ketone body synthesis, transferring one acetyl group to another acetyl-CoA (the crucial substrate for ketone body generation) [38]. Furthermore, the rate-limiting enzyme in ketone body synthesis is *HMGCS1*, which promotes β-hydroxy-butyrate production [39]. The increased expression of both *ACAT1* and *HMGCS1* in CS-fed cows may reflect an increased level of mammary ketone body production in cows consuming CS, which results in an increased level of ketone body utilization as energy for the mammary gland of these cows, in line with the high abundance of β-hydroxybutyric acid and acetoacetyl-CoA in blood of CS-fed cows versus AH-fed cows [7].

Amino acids are fundamental proteogenic substrates for milk synthesis in dairy cows, most of which derive from dietary proteins. After digestion and absorption in the small intestine, amino acids are taken up from the blood by the mammary gland via amino acid transporters. The protein *SLC7A8* is a system L amino acid transporter that functions by a Na^+^-independent and electroneutral transport mechanism for neutral amino acids [40]. In this study, mammary expression of SLC7A8 genes was 0.38- fold lower in CS-fed cows compared to the AH-fed cows, indicating a reduced system L amino acid transporter activity for neutral amino acids (including isoleucine, leucine, methionine, phenylalanine, threonine, alanine and serine) in the mammary gland of CS-fed cows [41]. This finding was in consistent with our previous observations of amino acid uptake in these cows [6, 31].

Ribosomes consist of a small 40S and a large 60S subunits, and ribosomal proteins are required for different stages of ribosome biogenesis and/or for distinct steps of the translation process [42]. All six ribosomal protein subunits (*RPS2*, *RPS12*, *RPS16*, *RPS19*, *RPS20* and *RPS27*) expressed at lower levels in CS-fed cows were essential components of the small ribosomal subunit (40S). The lower abundance of these ribosomal proteins in the CS group suggests depressed efficiency of protein translation, contributing to lower milk protein in these cows [31].

For protein processing, proteins and peptides must be transported into the endoplasmic reticulum (ER) or Golgi, where they acquire modifications that allow them to be biologically active [43]. Specific three-dimensional conformations acquired through folding of newly translated polypeptides and/or refolding of unfolded proteins are essential for protein function formation and maintenance [44]. The protein *DNAJB1* is a heat shock protein (HSP) [an ER molecular chaperone that can protect other proteins against occurrence of incorrect folding, but may also stimulate ER-associated degradation (ERAD)] [45]. As an ER molecular chaperone, the protein *DNAJB11* is essential to prevent the processes such as protein aggregation, allowing protein folding and assembly to proceed correctly [46]. In this study, expression of DNAJB1 and DNAJB11 was higher at the mRNA and protein levels in the CS-fed cows, suggesting that these cows may have higher degree of protein non-aggregation and protein degradation in the mammary gland. The protein heat shock 105 kDa/110 kDa protein 1 (*HSPH1*), another HSP, can be induced by several kinds of environmental stress [47] and is associated with mammary tumor tissues [48]. In the current study, the highly levels of HSPH1 in the CS-fed cows may suggest that the CS diet induces ER-stress in the mammary gland. The increased amount of protein degradation in the mammary gland is in accordance with the reduced milk protein and lactation performance in the CS group [6].

The proteasome is a large, 26S-multicatalytic protease that degrades poly-ubiquitinated proteins to small peptides [49]. The proteasome is composed of two sub-complexes: a core catalytic 20S particle and a regulatory 19S particle [50]. Expression of two 19S regulatory particles [proteasome 26S subunit, ATPase 6 (*PSMC6*) and proteasome 26S subunit, ATPase 2 (*PSMC2*)], the two 20S core particles [proteasome subunit alpha 3 (*PSMA3*) and proteasome subunit alpha 2 (*PSMA2*)] and proteasome 26S subunit, non-ATPase 14 (*PSMD14*) was higher in the mammary gland of cows fed the CS-based diet, supporting enhanced protein degradation in these cows. In addition, many intracellular proteins become covalently modified with ubiquitin (UB) or ubiquitin-like proteins (UBLs) [51]. Then, ubiquitin-conjugating enzymes (E1s) transfers the activated modifier to a family of E2 ubiquitin-conjugating enzymes, leading to their degradation [52]. In this study, expression of ubiquitin-conjugating enzyme 2B (*UBE2B*), ubiquitin-conjugating enzyme E2H (*UBE2H*) and target recognizing subunit kelch-like family member 9 (*KLHL9*) was higher in CS-fed cows, further indicating active protein degradation in the mammary glands of these animals. Taken together, the higher abundance of the proteasome-related and ubiquitin-dependent proteins is consistent with a higher degree of protein degradation in the mammary cells of CS-fed cows. Both the possibly increased protein degradation and reduced protein synthesis may result in lower milk protein yield in the CS group.

Peroxisomes are essential organelles that play a key role in lipid homeostasis. The protein SCP2 acts in peroxisome cholesterol transport through the cytoplasm, and loss of SCP2 can result in defects in fatty acid β-oxidation [53]. Higher SCP2 measured at mRNA and protein levels in cows fed CS-based diet suggested an enhanced level of fatty acid β-oxidation in the mammary gland. The protein isocitrate dehydrogenase [NADP] 1 (*IDH1*) is an enzyme that catalyzes oxidative decarboxylation of isocitrate, producing alpha-ketoglutarate (α-ketoglutarate) and CO_2_. Cytosolic IDH1 (EC 1.1.1.41) and mitochondrial IDH2 (EC 1.1.1.42) catalyze the same reaction outside the context of the citric acid cycle and use NADP^+^ as a cofactor instead of NAD^+^ [54]. Cytosolic IDH1 plays a complementary role in reductive glutamine metabolism, possibly through its oxidative function in an IDH2/IDH1 shuttle that transfers high energy electrons in the form of NADPH from mitochondria to cytosol, especially under hypoxia [55]. In this study, the higher IDH1 and the lower levels of IDH2 in CS-fed cows may indicate that the complementary IDH1-dependent carboxylation pathway is enhanced in CS-fed cows, enabling higher citrate production for energy supply. Acyl-CoA dehydrogenases (ACADs) are a class of enzymes that function to catalyze the initial step in each cycle of fatty acid β-oxidation in the mitochondria of cells [56]. Higher expression of acyl-CoA dehydrogenase (*ACADM*), combined with the higher levels of long fatty acid transporter (*SLC27A6*), in CS-fed cows may indicate a higher transport activity of long fatty acid from the plasma into the mammary cells, thus supporting a higher level of fatty acid oxidation for energy supply in these cows. Taken together, expression changes of multiple genes involved in fatty acid β-oxidation and fatty acid transport indicate a more active fatty acid metabolism to supply energy in the CS-fed cows compared to AH-fed cows. This observation is consistent with our previous finding that the CS-fed cows had a lower supply of acetate which is the major energy source in ruminants [6].

Finally, six collagen proteins and five genes encoding collagen proteins were lower in the mammary gland of CS-fed cows. Collagens are the main structural proteins in extracellular matrix (ECM), and are important regulators of the differentiated phenotype of mammary epithelial cells in culture [31, 32]. Lower levels of these proteins may indicate a different mammary gland morphology in these animals. Specifically, a decrease in the levels of the protein COL4A2 was found to be associated with a loss of basement membrane integrity, accompanied by a dramatic alteration of alveolar morphology with decreased size and shrunken lumen containing little β-casein [32]. Mammary structural changes may be another factor contributing to the reduction of milk yield in the CS-fed cows compared with cows fed AH [31].

## Conclusions

By integrating transcriptomic and proteomic data, this study suggested four major possible mechanisms contributing to the lower milk production in dairy cows fed CS-diet compared to AH-diet: (i) reduced mammary growth/development through lower expression of *COL4A2*, *MAPK3*, *IKBKB,* and *LGALS3*, (ii) less oxidative phosphorylation through lower expression of *ATPsynGL*, *ATP6VOA1*, *ATP5H*, *ATP6VOD1,* and *NDUFC1* with enhanced lipid uptake and fatty acid beta-oxidation to supply energy through higher expression of *SLC27A6*, *FABP4*, *SOD2*, *ACADM*, *ACAT1*, *IDH1*, *SCP2*, *ECHDC1,* and *HMGCS1*, (iii) less AA/protein transport and metabolism through lower expression of *SLC38A2*, *SLC7A8*, *RAB5a,* and *VPS18*, and less protein translation through lower expression of *RPS6*, *RPS12*, *RPS16*, *RPS19*, *RPS20,* and *RPS27*, and (iv) more proteasome- and ubiquitin-mediated protein degradation through higher expression of the protease components *PSMC2*, *PSMC6*, *PSMD14*, *PSMA2*, *PSMA3*, and ubiquitin-conjugating enzymes *UBE2B*, *UBE2H*, *KLHL9*, *HSPH1*, *DNAJA1,* and *CACYBP*, and protein disassembly-associated enzymes *SEC63*, *DNAJC3*, *DNAJB1*, *DNAJB11,* and *DNAJC12*. These mechanisms involved in milk production of dairy cows fed CS-forage-based diet can direct future work to further understand how the mammary gland adapts to low nutrient availability and, ultimately, to change feeding strategies so that cows can utilize low-quality forage more efficiently.

## Additional files

Additional file 1: **Table S1.** Primers used in real-time RT-PCR. (XLSX 11 kb)

Additional file 2: **Table S2.** Summary of the transcriptome data in the mammary gland of dairy cows fed corn stover (CS) or alfalfa hay (AH). **Table S3.** Summary of the proteome data in the mammary gland of dairy cows fed corn stover (CS) or alfalfa hay (AH)). (ZIP 13 kb)

Additional file 3: **Table S4.** Differentially expressed genes in the mammary gland of cows fed corn stover (CS) vs. alfalfa hay (AH). The cutoff is set at 1.5-fold change and *p* < 0.05. **Table S5.** Differentially expressed proteins in the mammary gland of cows fed corn stover (CS) vs. alfalfa hay (AH). The cutoff is set at 1.2-fold change and *p* < 0.05. **Table S6.** Differentially expressed genes found in both transcriptomic and proteomic analyses in the mammary gland of cows fed corn stover (CS) vs. alfalfa hay (AH). (ZIP 164 kb)

Additional file 4: **Table S7.** A list of common differentially expressed genes and differentially expressed proteins in the mammary gland of cows fed corn stover (CS) vs. alfalfa hay (AH). (XLSX 17 kb)

Additional file 5: **Table S8.** The full name and abbreviation of proteins listed in Figs. 6 and 7. (XLSX 14 kb)

Additional file 6: **Table S9.** The full name and abbreviation of proteins in Fig. 8. (XLSX 16 kb)

## Abbreviations

*AARS2*: Probable alanyl-tRNA synthetase
*ACADM*: Acyl-CoA dehydrogenase, C-4 to C-12 straight chain
*ACAT1*: Acetyl-CoA acetyltransferase
*ACN*: Acetonitrile
*ACSS1*: Acetyl-coenzyme A synthetase 1
*AGC*: Automatic gain control
*AH*: Alfalfa hay based diet
*ATP5H*: ATP synthase subunit d
*ATP6V0A1*: Isoform 1 of V-type proton ATPase 116 kDa subunit a isoform 1
*ATP6V0D1*: V-type proton ATPase subunit d1
*ATPsynGL*: ATP synthase, H+ transporting, mitochondrial F0 complex, subunit G-like
*CACYBP*: Calcyclin binding protein
*CHAPS*: 3-[(3-Cholamidopropy) dimethylammonio] propane-sulfonate
*COL4A2*: Collagen, type IV, alpha 2
*CS*: Corn stover based diet
*DBT*: Dihydrolipoamide branched chain transacylase E2
*DMGDH*: Dimethylglycine dehydrogenase
*DNAJA1*: DnaJ (Hsp40) homolog, subfamily A, member 1
*DNAJB1*: DnaJ (Hsp40) homolog, subfamily B, member 1
*DNAJB11*: DnaJ homolog subfamily B member 11
*DNAJC12*: DnaJ homolog subfamily C member 12
*DNAJC3*: DnaJ homolog subfamily C member 3
*ECHDC1*: Enoyl CoA hydratase domain containing 1
*FA*: Formic acid
*FABP4*: Fatty acid binding protein 4
*FDR*: False discovery rate
*GAPDH*: Glyceraldehyde-3-phosphate dehydrogenase
*HK1*: Hexokinase 1
*HMGCS1*: Hydroxymethylglutaryl-CoA synthase
*HSPH*: Heat shock 105 kDa/110 kDa protein 1
*HSPH1*: Heat shock 105 kDa/110 kDa protein 1
*IDH1*: Isocitrate dehydrogenase [NADP]
*IDH2*: Isocitrate dehydrogenase [NADP]
*IKBKB*: Inhibitor of kappa light polypeptide gene enhancer in B-cells, kinase beta
*KLHL9*: Kelch-like family member 9
*LGALS3*: Galectin-3-binding protein
*MAOA*: Amine oxidase [flavin-containing] A
*MAP K3*: Mitogen-activated protein kinase 3
*NDUFC1*: NADH dehydrogenase [ubiquinone] 1 subunit C1
*PDHX*: Pyruvate dehydrogenase complex, component X
*PFKP*: Phosphofructokinase
*POLR1A*: Polymerase (RNA) I polypeptide A, 194 kDa
*POLR1B*: Polymerase (RNA) I polypeptide B, 128 kDa
*POLR2B*: DNA-directed RNA polymerase
*POMP*: Proteasome maturation protein
*PSMA2*: Proteasome subunit alpha 2
*PSMA3*: Proteasome subunit alpha 3
*PSMC2*: Proteasome 26S subunit, ATPase 2
*PSMC6*: Proteasome 26S subunit, ATPase 6
*PSMD14*: Proteasome 26S subunit, non-ATPase 14
*PYGB*: Phosphorylase, glycogen
*RAB5a*: Ras-related protein Rab-5A
*RPS12*: 40S ribosomal protein S12
*RPS16*: 40S ribosomal protein S16
*RPS19*: 40S ribosomal protein S19
*RPS2*: 40S ribosomal protein S2
*RPS20*: 40S ribosomal protein S20
*RPS27*: 40S ribosomal protein S27
*SARDH*: Sarcosine dehydrogenase
*SCP2*: Sterol carrier protein 2
*SCX*: Strong cationic exchange
*SEC63*: SEC63 homolog, protein translocation regulator
*SLC27A6*: Solute carrier family 27 (fatty acid transporter), member 6
*SLC38A2*: Solute carrier family 38, member 2
*SLC7A8*: Solute carrier family 7 (amino acid transporter light chain, L system), member 8
*SOD2*: Superoxide dismutase [Mn]
*SUCLG1*: Succinyl-CoA ligase [GDP-forming] subunit alpha
*TARS2*: Threonyl-tRNA synthetase 2
*TEAB*: Triethylamine borane
*UBE2B*: Ubiquitin-conjugating enzyme E2B
*UBE2H*: Ubiquitin-conjugating enzyme E2H
*VPS18*: VPS18 protein
